# Effects of Open-Label Placebos on Visual Food Cue Reactivity in Children and Adolescents

**DOI:** 10.3390/children11111320

**Published:** 2024-10-30

**Authors:** Anne Schienle, Alice Polz, Katharina Haslacher, Florian Osmani, Wolfgang Kogler

**Affiliations:** Clinical Psychology, University of Graz, 8010 Graz, Austria

**Keywords:** open-label placebo, children, adolescents, appetite, food images

## Abstract

Background: A high level of food cue reactivity (FCR) is a risk factor for overeating and weight gain. This randomized trial investigated whether open-label placebos (OLPs: placebos honestly administered) can reduce FCR (reported appetite) in children and adolescents. Method: Children (*n* = 73; 8–12 years old) and adolescents (*n* = 187; 16–18 years old) were randomly assigned to either an OLP group or a control group (without OLP). Participants viewed images depicting food (sweets and fruits) and non-food items. Before viewing, the OLP group received a placebo for appetite reduction. Participants rated their desire to eat the depicted food items (0–100) and the perceived effectiveness of the OLP intervention. Results: The OLP exhibited a large effect in children, leading to a general reduction in appetite (group difference OLP vs. no OLP: M = −20.8; ηp2 = 0.20). This general effect was absent in adolescents, whose appetite reduction was restricted to fruits (OLP vs. no OLP: M = −8.1; ηp2 = 0.03). Additionally, children perceived the OLP as more effective than adolescents. Conclusion: The reduced response and skeptical attitudes of adolescents towards OLP treatment require further investigation.

## 1. Introduction

Food cue reactivity (FCR) refers to how individuals respond to food-related stimuli, such as the sight, smell, or even the thought of food. Both adults and children with high levels of FCR are more prone to overeating, which increases their risk of developing overweight and obesity [1,2]. Lifestyle changes, including healthier eating habits (e.g., reducing sugar intake) and increasing physical activity, are effective strategies for preventing and treating obesity [3]. However, motivation for these behavioral changes and long-term adherence are often insufficient. Thus, there is a need for simple, easy-to-administer methods that can modify FCR and complement established approaches for appetite and weight control.

One such method involves placebos, which can be defined as treatments that elicit positive outcomes not related to active treatment components but to positive expectations, learning experiences, and/or the psychosocial context in which the treatment takes place [4]. Placebos can be administered deceptively (e.g., as ‘appetite suppressants’) or with full transparency as open-label placebos (OLPs). Both types of placebo treatment have been associated with appetite reduction in adults as demonstrated in various clinical trials [5,6] and studies involving both healthy, normal-weight individuals and those who are overweight [7,8,9,10,11,12].

In the studies mentioned, placebo intake was associated with reported mood [10] and attentional processes [9]. In the latter experiment, which used eye-tracking, participants showed fewer fixations on food images while under placebo treatment. A recent study [11] demonstrated that a 4-week OLP treatment reduced reported hunger and increased perceived cognitive control of eating in a group of overweight adults. These findings show that placebos can exert their effects concerning FCR by altering emotional states, the visual exploration of food cues, and cognitive control.

Placebo studies with pediatric samples remain relatively scarce, even though research has suggested that placebo response rates often tend to be higher in children compared to adults (as reviewed by [13]). Further, the effects of OLPs have been especially underexplored in children and adolescents. This is surprising since OLPs can have beneficial effects in clinical conditions characterized by negative affectivity and reduced emotional and/or behavioral control (e.g., depression, anxiety disorders, and attention-deficit hyperactivity disorder), as well as in non-clinical conditions (emotional distress; see meta-analyses by [14,15]). OLPs have been shown to improve emotion regulation [16] and cognitive control [11].

To date, only three studies have been conducted that have looked at the effects of OLPs in children/adolescents [17,18,19]. These studies comprised participants diagnosed with attention-deficit hyperactivity disorder, functional abdominal pain, or irritable bowel syndrome. The participants in these studies were also concurrently receiving pharmacotherapy in the form of stimulants or spasmolytics. The OLP treatment was found to reduce medication intake [17,18,19] and reported pain [19]. Furthermore, the majority of these participants found the placebos to be beneficial [18], and there were no reported adverse treatment effects [19].

The current study contributes to existing research on OLPs by examining their influence on the reported desire to eat in two age groups: children (aged 8 to 12 years, as explored in Study 1) and adolescents (aged 16 to 18 years, as explored in Study 2). The desire to eat a specific food together with its liking (perceived pleasantness) are two major forces that drive eating behavior in humans [20].

The present study was conducted in school settings within classroom environments. Participants were randomly assigned to one of two groups (OLP or Control). Both groups were exposed to images depicting food items (high-calorie or low-calorie) as well as non-food (control) items. Each food image was assessed for liking and desire to eat by the participants. Before viewing the images, the OLP group received an OLP (‘an oral spray for appetite reduction’), whereas the Control group received no placebo treatment. It was predicted that the placebo would reduce the reported desire to eat the depicted food items [9,11]. In addition, participants rated the perceived effectiveness of the intervention. After the experiment, they had the opportunity to give open comments about the OLP treatment.

## 2. Materials and Methods

### 2.1. Participants

Study 1 (Children): A total of 73 children (mean age = 9.62 years, SD = 1.4; 51% female) were randomly assigned (random number table) to the OLP group (*n* = 37) or the Control group (*n* = 36). The two groups did not differ in mean age (M_OLP_ = 9.40 years, SD = 1.28; M_Control_ = 9.91 years, SD = 1.53; t(51) = 1.33, *p* = 0.19) and body mass index (MOLP = 16.6, SD = 3.3; M_Control_ = 18.2, SD = 3.3; t(50) = 1.75, *p* = 0.09). The Control group reported a higher baseline appetite level than the OLP group (M_OLP_ = 46.0, SD = 29.3; M_Control_ = 64.7, SD = 29.4; t(71) = 2.73, *p* = 0.008).

Study 2 (Adolescents): A total of 187 high school students (mean age = 16.90 years, SD = 0.66; 77% female) were randomly assigned to the OLP group (*n* = 93) or the Control group (*n* = 94). The two groups did not differ in body mass index (M_OLP_ = 21.7, SD = 3.2; M_Control_ = 21.3, SD = 2.4; t(184) = 1.02, *p* = 0.31) and reported appetite level at baseline (M_OLP_ = 49.72, SD = 26.42; M_Control_ = 45.05, SD = 23.28; t(185) = 1.28, *p* = 0.20). Adolescents in the Control group were slightly older than those in the OLP group (M_OLP_ = 16.8 years, SD = 0.7; M_Control_ = 17.1 years, SD = 0.6, t(185) = 2.83, *p* = 0.005).

A priori power analyses were conducted with G*Power [21] separately for children and adolescents. These analyses showed that, for two groups (OLP and Control) and four picture types (sweets, fruits, school supplies, and animals) and assumed effect sizes of 0.16 (for children; partial eta2 = 0.025) and 0.12 (for adolescents; partial eta2 = 0.014) together with an alpha error probability of 0.05 and a power of 0.80, 56 children and 98 adolescents would be needed for the studies. The effect sizes were based on the placebo literature, which indicates strong placebo effects in children (e.g., [13]).

The recruitment of participants was accomplished by directly contacting the schools (principals and teachers). The only inclusion criterion for the study was the age range of the participants: 8–12 years (children); and 16–18 years (adolescents). We obtained larger sample sizes as intended because all students who agreed to participate were tested (see CONSORT diagram; Supplementary Figure S1).

### 2.2. Images

**Figure 1:**
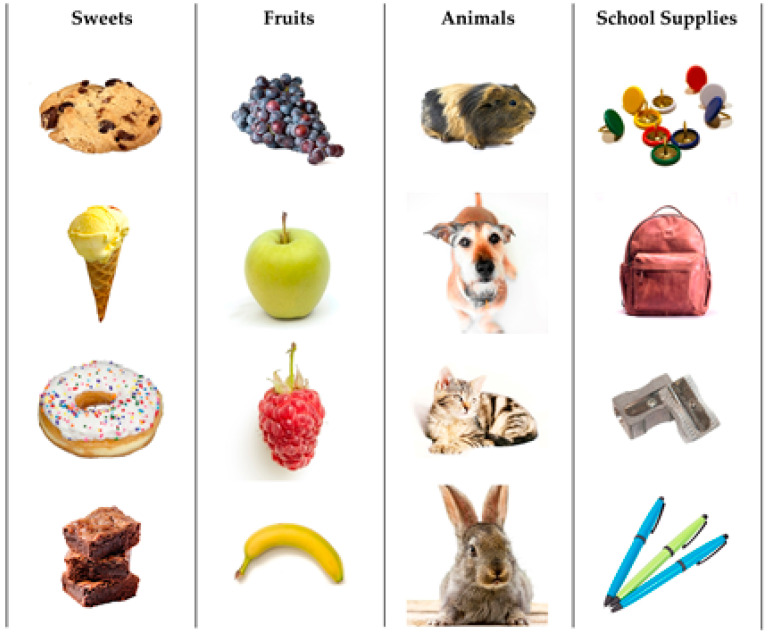
Images.

All participants viewed a total of 16 images from four categories (high-calorie sweets, low-calorie fruits, school supplies, and cute animals). Each food/non-food category comprised four images (Figure 1). The pictures were taken from a validated picture set (Food-Pics, [22]) and free media platforms (Pixabay and Unsplash). The images of each category were matched according to physical features such as brightness, color composition, and complexity.

Each image was shown for 6 s. In Study 1 (children), the pictures were projected onto the wall of the classroom. In Study 2 (adolescents), the image presentation was carried out via a smartphone application (participants used their smartphones). The pictures were presented in a randomized sequence.

Each image was rated according to liking (‘How much do you like this?’; 0 = not at all, and 100 = very much). The food images were additionally rated according to desire to eat (‘Would you like to eat this right now?’; 0 = not at all, and 100 = very much).

### 2.3. Design and Procedure

This randomized controlled trial had a parallel design. Participants were either assigned to the OLP group or the Control group (no OLP). Since the testing was conducted in the classrooms, all students of a class received the same treatment.

The study was conducted between September 2023 and January 2024 at 11 different schools (5 elementary schools, and 6 high schools) always in the mornings (before lunch time). The study was approved by the Ethics Committee of the University of Graz (GZ. 39/L57/63 ex 2022/23; approval date: 1 September 2023) and the school directory. The procedure followed the Declaration of Helsinki. Adolescents, and parents/caregivers of the children provided written informed consent as well as information about the weight and height of their children (22 parents agreed to the participation of their children but did not provide information about their height/weight). The study was preregistered on the Open Science Framework (OSF https://osf.io/udf8j) and additionally registered on the German Clinical Trial Register (https://www.drks.de/DRKS00035122).

The testing was performed in groups by two experimenters. All participants first rated their appetite level (baseline) on a scale ranging from 0 = no appetite to 100 = strong appetite. Then, participants were provided with information about placebos and assumed placebo mechanisms (expectations, learning, and context factors) via a PowerPoint presentation. Two versions of this presentation were created, one for children and one for adolescents (Supplementary Figures S2 and S3).

After the presentation, the experiment and the rating procedure for the images were explained. For the ratings, the children received a rating booklet. Each page of the booklet showed a small version of the image as well as the visual analog scales for the ratings (liking/desire to eat). When each child in a group indicated the completion of the rating, the image presentation was continued. Adolescents were instructed how to rate the images on visual analog scales (sliders) via a smartphone application (no time limit for the ratings; after a rating, the next picture was shown).

After this, each OLP participant received a placebo spray bottle with the label “Placebo—for less appetite” with the information “This is a 30 mL bottle filled with blue-colored water”. The placebo spray was administered orally (three pumps). The OLP administration was accompanied by the verbal suggestion that the placebo can reduce appetite.

Participants of the Control group did not receive the OLP before the picture presentation. They were informed that, first, a baseline assessment would be conducted. After having viewed and evaluated the 16 images, the Control group received the placebo spray. Four additional pictures (one for every picture category) were shown and rated (these ratings were not included in the analysis). This procedure was chosen since the time for the experiment was restricted to one hour (one school lesson).

The participants were seated at a sufficient distance from each other, so they were not able to see the answers of the other participants. They were explicitly instructed not to talk to each other during the experiment. After finishing all picture ratings, the perceived effectiveness of the OLP was rated on a scale ranging from 0 (not at all effective) to 100 (very effective) either with the booklet (children) or the smartphone application (adolescents).

Directly after the experiment, participants were encouraged to raise any questions related to the experiment and to provide feedback. Open comments of the students are listed in Supplementary Table S1. After completing the study, each class received written information summarizing the main findings of the experiment.

### 2.4. Statistical Analysis

An analysis of covariance (ANCOVA) was computed to test the effects of Group (OLP or Control) and Picture Type (sweets or fruits) on the reported desire to eat the depicted food items by adjusting for reported baseline appetite (covariate). An analysis of variance (ANOVA) was computed to test the effects of Group (OLP or Control) and Picture Type (sweets, fruits, school supplies, or animals) on the ratings for liking the depicted items.

Due to the differences in sample sizes, and age-adapted presentations, the analyses were separately conducted for children and adolescents. Significant effects were followed up via *t*-tests (with Bonferroni correction).

## 3. Results

### 3.1. Study 1 (Children)

**Figure 2:**
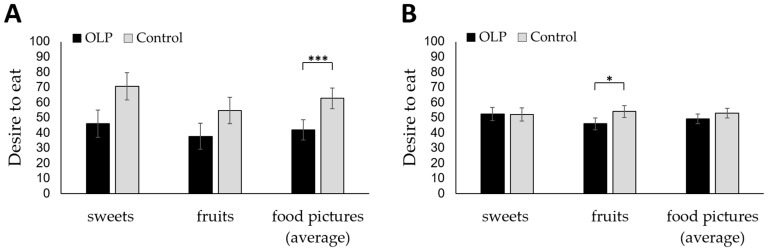
Reported desire to eat the depicted food items. Ratings for desire to eat (adjusted for baseline appetite) in the open-label placebo (OLP) group and the Control group in children (**A**) and adolescents (**B**). *** *p* < 0.001; * *p* < 0.05. Error bars depict 95% CI.

Desire to eat: The main effect for Group was significant (F(1,70) = 17.63, *p* < 0.001, ηp2 = 0.20). The OLP group (M_adjusted_ = 41.90, SD = 20.68) reported less desire to eat the depicted food items than the control group (M_adjusted_ = 62.70, SD = 20.70; Figure 2). The effect for Baseline Appetite (covariate) was significant (F(1,70) = 8.65, *p* < 0.01, ηp2 = 0.11). The main effect for Picture Type (F(1,70) = 1.37, *p* = 0.25, ηp2 = 0.02), the interaction Group x Picture Type (F(1,70) = 0.85, *p* = 0.36, ηp2 = 0.01), and the interaction Baseline Appetite x Picture Type (F(1,70) = 0.08, *p* = 0.78, ηp2 = 0.001) were not significant.

Liking: The main effect for Picture Type was significant (F(3,201) = 116.68, *p* < 0.001, ηp2 = 0.64). The reported liking for school supplies was lower than those for all other picture types (all p_bonf_ < 0.001, see Table 1). Furthermore, animals received higher ratings than fruits (t(67) = 3.92, p_bonf_ = 0.001, d = 0.48). The effects for Group (F(1,67) = 1.34, *p* = 0.25, ηp2 = 0.02) and the interaction Group x Picture Type (F(3,201) = 1.78, *p* = 0.15, ηp2 = 0.03) were not significant.

### 3.2. Study 2 (Adolescents)

Desire to eat: There was a significant interaction Group x Picture Type (F(1,183) = 5.22, *p* = 0.02, ηp2 = 0.03). The OLP group (M_adjusted_ = 45.90, SD = 19.20) reported less desire to eat fruits than the Control group (M_adjusted_ = 54.00, SD = 21.5); t(183) = 2.96, pbonf = 0.02, mean difference = 8.10). There was no significant group difference in the desire to eat sweets (OLP: M_adjusted_ = 52.30, SD = 21.40; control: M_adjusted_ = 52.00, SD = 23.90; t(183) = 0.11, pbonf = 0.99, mean difference = 0.30; see Table 1). The effect for Baseline Appetite (covariate) was significant (F(1,183) = 46.48, *p* < 0.001, ηp2 = 0.20). The main effects for Group (F(1,183) = 2.82, *p* = 0.10, ηp2 = 0.02) and Picture Type (F(1,183) = 0.92, *p* = 0.34, ηp2 = 0.005) as well as the interaction Baseline Appetite x Picture Type (F(1,183) = 0.22, *p* = 0.64, ηp2 = 0.001) were not significant.

Liking: The main effect for Picture Type was significant (F(3,555) = 265.11, *p* < 0.001, ηp2 = 0.59). Ratings for school supplies were lower than those for the other picture types (all p_bonf_ < 0.001, see Table 1). Furthermore, ratings were higher for animals compared to fruits (t(185) = 7.60, p_bonf_ < 0.001, d = 0.56) and sweets (t(185) = 4.70, p_bonf_ < 0.001, d = 0.34). The effects for Group (F(1,185) = 0.095, *p* = 0.76, ηp2 = 0.001) and the interaction Group x Picture Type (F(3,555) = 0.73, *p* = 0.54, ηp2 = 0.004) were not significant.

### 3.3. Perceived Effectiveness of the Treatment

Children rated the effectiveness of the OLP as average (M = 56.1, SD = 35.1; range = 0–100). Adolescents gave lower effectiveness ratings (M = 27.7, SD = 23.2; range = 0–100; t(258) = 7.60, *p* < 0.001, d = 1.05) than children.

### 3.4. Open Comments About the OLP Treatment

Children: The experimenters noted 18 open comments by OLP participants after the experiment—12 of them were positive (e.g., ‘“I’m not hungry at all anymore”, and “It worked super-duper for me”). The remaining comments were negative (“I am hungry, that didn’t help at all”, and “I didn’t notice anything at all”) or neutral (“I was generally not hungry”).

Adolescents: The experimenters noted 14 comments from OLP participants. One comment was positive (‘I believe it helped me”). Two students reported having used placebos before to treat their anxiety symptoms (test anxiety and panic attacks) but found the OLP in the present study not to be helpful. Two students stated that the OLP even increased their desire to eat something, and another two questioned the OLP concept: “I don’t believe something like that works, because it’s stupid to tell us beforehand” and “I can’t imagine that it works; but if I didn’t know there was nothing in it [the placebo], then it would surely work much better.” All comments can be found in Supplementary Table S1.

## 4. Discussion

This study aimed to investigate whether an OLP, administered before the viewing of food images, could influence food cue reactivity (FCR) in children and adolescents. The results indicated that children in the OLP group reported less desire to eat the depicted food items compared to the Control group without a placebo. This decrease was observed for both high-calorie and low-calorie items, suggesting a general placebo-associated reduction in food wanting. The effect size was large. These findings align with previous research on placebo effects in children, demonstrating the responsiveness of this age group to such treatments (e.g., [17,18,19,23]).

Adolescents only reported a reduced desire to eat fruits due to OLP treatment, while the appetite for sweets did not differ between the placebo and control groups. Additionally, adolescents rated the effectiveness of the OLP lower compared to children. Research with adults has already documented that the OLP concept can be subject to skepticism (e.g., [24,25,26,27]). Some of the study participants view the oral administration of a substance recognized as inert as an implausible procedure. A recent survey of adults [28] found polarized attitudes to OLP treatment. Approximately one-third of the respondents indicated that they would never use an OLP themselves or give one to their child, neither for the management of somatic symptoms nor emotional distress. In a qualitative study using a thematic analysis of semi-structured interviews by [25], participants mostly conceptualized a placebo as something that requires deception to be effective, a view that was also expressed by some of the adolescent participants in their open comments. Finally, in a survey with pediatric patients (age range: 12–18 years), only 55% of the respondents indicated that they would be willing to take an OLP for two weeks to explore if symptom reduction would occur [29]. This points to skeptical attitudes in half of the group with a similar age range as in the present investigation.

It is noteworthy that the present study protocol included a brief presentation on placebos as well as their assumed mechanisms before the experiment (based on an expert consensus paper by [30]). Moreover, a rationale was provided, which previously has been associated with both the acceptance of placebo treatment and positive outcomes (e.g., [31]). However, despite this approach, skepticism among some adolescent participants persisted, as evidenced by their open comments. It is important to find out more about the specific reasons for this skepticism in a future investigation and ways to counteract these negative attitudes. Prior OLP studies have found that both the structure and content of OLP interventions modulate their success (e.g., [31,32,33]).

In contrast, children were less skeptical. This cannot be attributed to a misunderstanding of the OLP concept. The children were explicitly informed that the placebo spray they received contained only water. Moreover, we used practical examples from their own experiences to explain the OLP concept. For instance, during the instruction phase, we referred to the familiar practice of parents blowing on their wounds to ease the pain. The children understood—based on their verbal feedback—that this action has no physical effect but provides psychological and emotional comfort. The children reflected on OLPs quite elaborately, with comments such as, “You can tell that it helps, but it’s not really like that”. Thus, the placebo was perceived as a helpful agent to reduce appetite, even though the children were aware that water cannot have such effects. These findings are promising, indicating an understanding of the OLP concept even among young children. Moreover, in both age groups, the OLP did not affect the liking of the depicted items, indicating that the placebo specifically affected the desire to eat (as instructed).

This study has several potential limitations. We did not examine a representative group of children and adolescents but a self-selected sample with a predominance of females, reflecting the gender ratio in the school classes tested. Additionally, the present study relied on self-report measures (self-reported desire to eat) without assessing actual eating behavior or physiological parameters. This should be considered in a subsequent study. Surprisingly, the appetite-reducing OLP effect in adolescents was limited to fruits. Further studies should include a wider range of food categories to explore this effect more comprehensively. Introducing both high- and low-calorie foods as stimulus material would provide a more detailed understanding.

More broadly, although a non-deceptive placebo was used in this study, ethical complexities remain, particularly regarding informed consent in children. Since children require parental or caregiver consent to take an OLP, they do not have full autonomy. However, we prioritized the rights of the children by emphasizing that participation was voluntary and could be withdrawn at any time without consequences.

## 5. Conclusions

In conclusion, while the OLP showed efficacy in reducing appetite in children, it only had minor effects in adolescents. The effects might, however, be greater in clinical groups (e.g., children/adolescents with eating disorders) as suggested by a meta-analysis by [34].

## Figures and Tables

**Table 1:** Average ratings (standard deviations) for the images in the open-label placebo (OLP) group and the Control group.

	OLP M (SD)	Control M (SD)
**Adolescents: Liking**		
Sweets	62.2 (21.1)	60.5 (19.5)
Fruits	58.7 (17.6)	58.0 (18.5)
Animals	69.3 (18.6)	71.6 (16.4)
School supplies	25.5 (14.0)	27.7 (17.1)
**Adolescents: Desire to eat**		
Sweets	52.3 (21.4)	52.0 (23.9)
Fruits	45.9 (19.2)	54.0 (21.5)
**Children: Liking**		
Sweets	75.2 (24.5)	86.2 (12.7)
Fruits	73.4 (22.4)	76.4 (18.0)
Animals	83.7 (17.2)	85.2 (16.9)
School supplies	35.2 (19.0)	32.3 (19.6)
**Children: Desire to eat**		
Sweets	46.0 (30.5)	70.7 (24.7)
Fruits	37.7 (25.6)	54.7 (27.1)

## Data Availability

The data are available at https://osf.io/382vd/.

## Supplementary Materials

The following supporting information can be downloaded at: https://www.mdpi.com/article/10.3390/children11111320/s1, Figure S1: CONSORT diagrams, Figure S2: Presentation (for children), Figure S3: Presentation (for adolescents), Table S1: Open comments.
