# Supplementary material for: Effects of Open-Label Placebos on Visual Food Cue Reactivity in Children and Adolescents
Source: Children (Basel). 2024 Oct 30;11(11):1320. doi: 10.3390/children11111320 (PMC11592452; doi:10.3390/children11111320)
Supplement: Supplementary file 1 [file children-11-01320-s001.zip › children-3264598-supplementary.pdf]

Supplementary Figure S1: CONSORT diagrams for group 1 (children; top) and group 2 (adolescents; bottom)

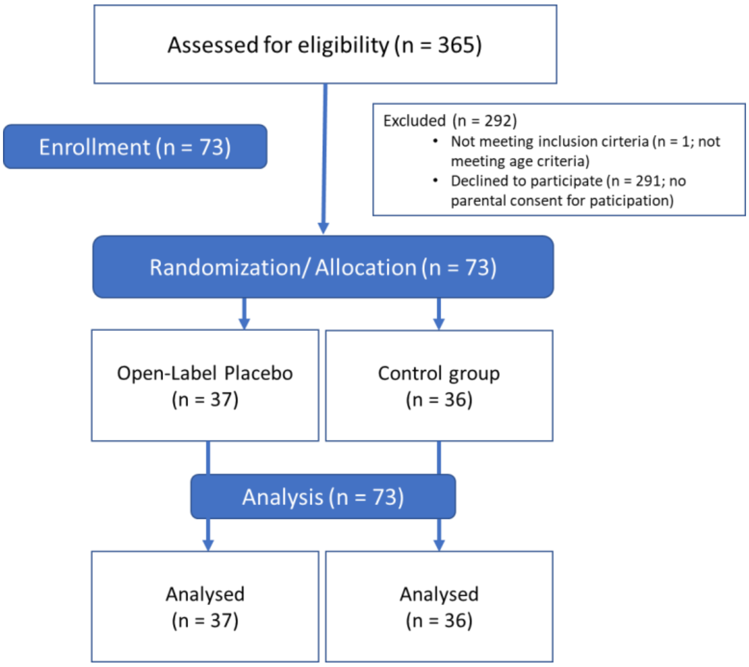

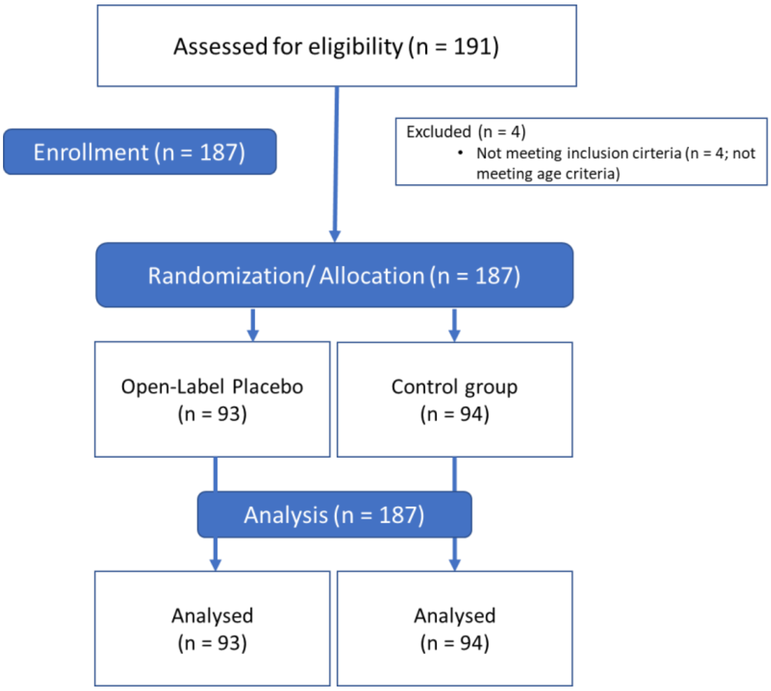

Supplementary Figure S2: Presentation (for children)

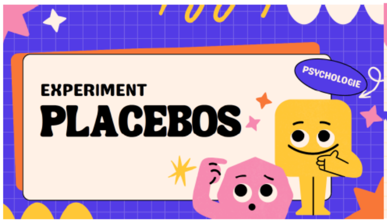

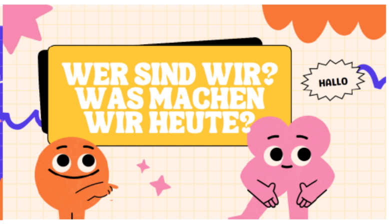

Experiment Placebos

Who are we?
What are we are going to do today?

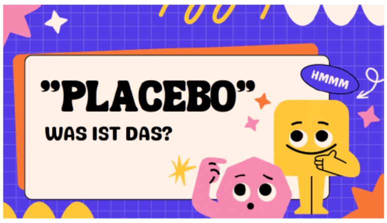

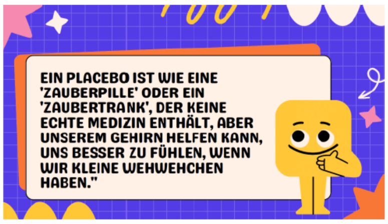

A placebo is like a 'magic pill' or a ' magic potion' with no real medication, but it can help the brain that we feel better when we have minor ailments.

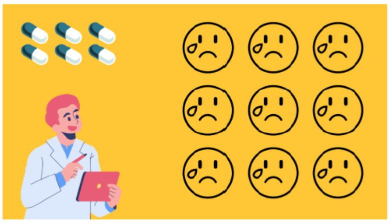

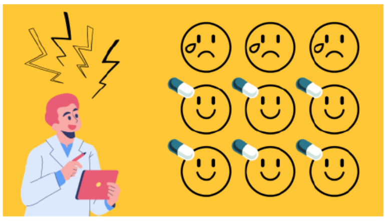

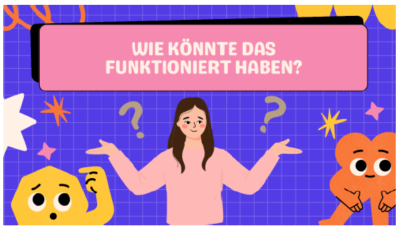

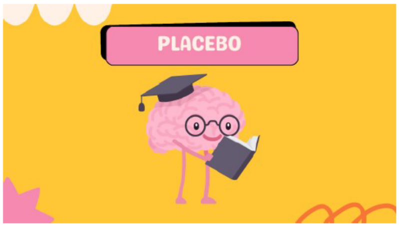

How could this function?

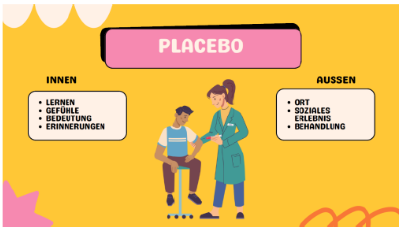

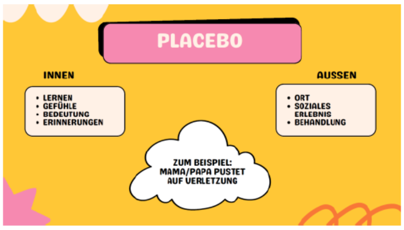

Inside:
Learning, Feelings, Meaning, Memories

Outside: Location, social encounter, treatment
for instance: mum/dad blow on your injury

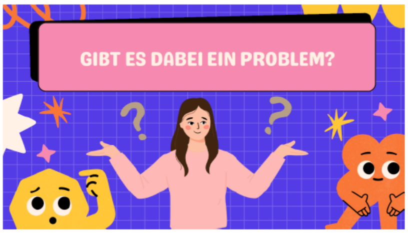

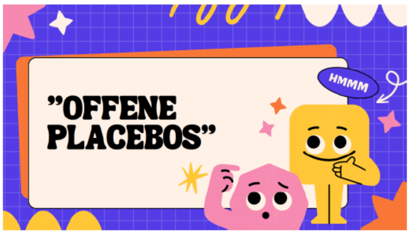

Is there a problem?

open placebos

Supplementary Figure S3: Presentation (for adolescents)

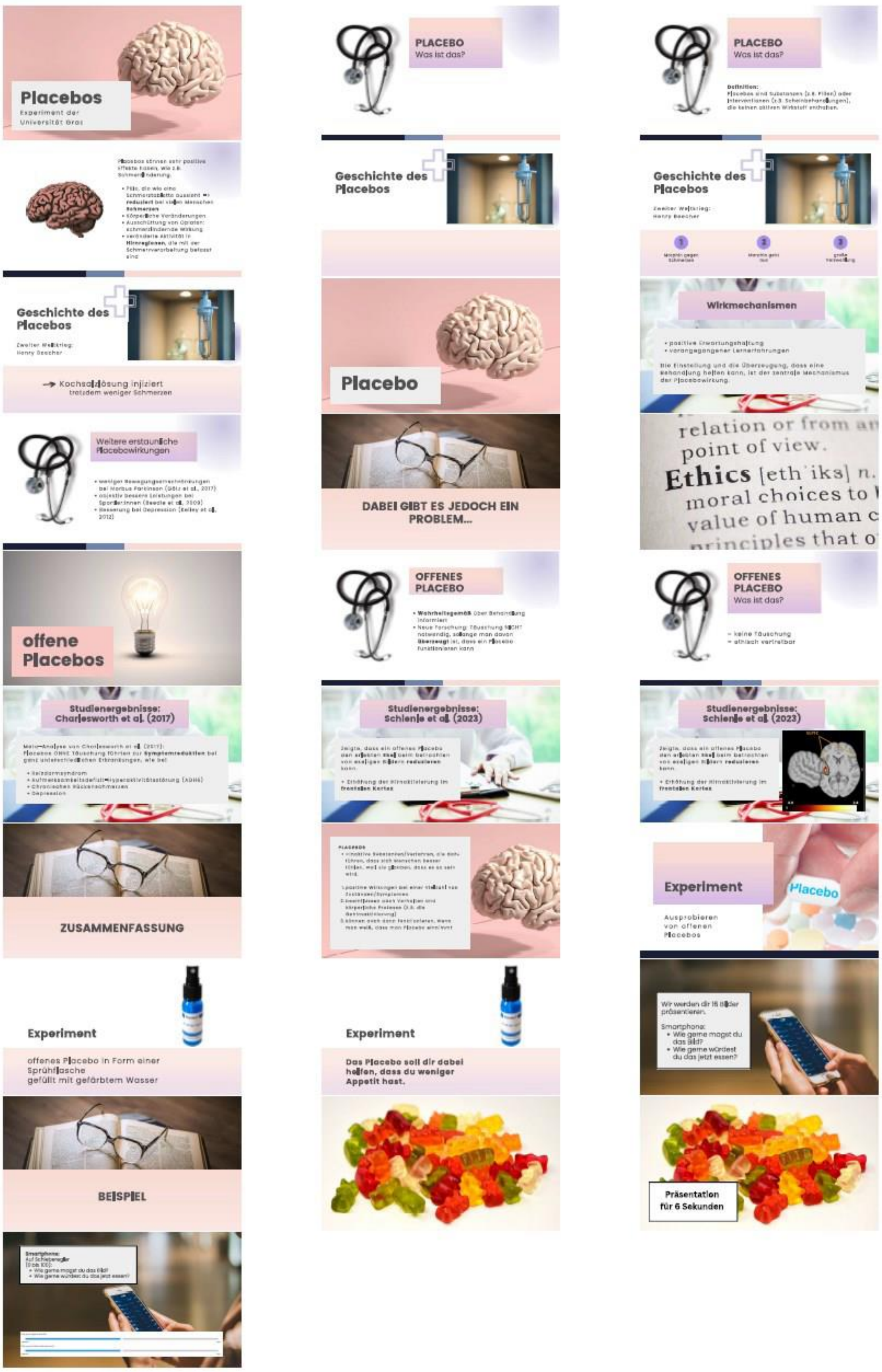

Translation

Slide 1 „Placebo - Experiment at the University of Graz"

S2 „Placebo – What ist that?“

S3 „Placebo – What ist that?“; „"Definition: Placebos are substances (e.g., pills) or interventions (e.g., sham treatments) that contain no active ingredient."

S4 „Placebos can have very positive effects, such as pain relief“; „A pill that looks like a painkiller -> reduces pain in many people“; „physical changes“; „release of opioids: painrelieving effect“; „altered activity in brain regions involved in pain processing“

S5 „History of the Placebo"

S6 „History of the Placebo"; „Second World War: Henry Beecher"; „-Morphine for pain relief“; Morphine runs out"; „Great despair"

S7„History of the Placebo"; „Second World War: Henry Beecher"; „-> Saline injected"; „still less pain"

S8 „Placebos“

S9 „Mechanisms of action“; „Positive expectations“; „Previous learning experiences“; „The attitude and belief that a treatment can help is the central mechanism of the placebo effect.“

S10 „Further remarkable placebo effects“; „Reduced movement limitations in Parkinson's disease (Götz et al., 2017)“; „Objectively better performance in athletes (Beedie et al., 2009)“; „Improvement in depression (Kelley et al., 2012)“

S11 „However, there is a problem...“

S12 …

S13 „Open Label Placebos“

S14 „Open Label Placebos“; „truthfully informed about the treatment“; „New research: Deception NOT necessary as long as one believes that a placebo can work"

S15 „Open Label Placebos - What ist that?“; „= no deception“; „= ethically justifiable“

S16 „Study Results: Charlesworth et al. (2017)“; „Meta-analysis by Charlesworth et al. (2017): Placebos WITHOUT deception led to symptom reduction in a variety of conditions, such as irritable bowel syndrome, attention deficit hyperactivity disorder (ADHD), chronic back pain, and depression“

S17 „Study Results: Schienle et al. (2023)“; „showed that an open-label placebo can reduce the experienced disgust when viewing disgusting images“; „+ increase in brain activity in the frontal cortex“

S18 „Study Results: Schienle et al. (2023)“; „showed that an open-label placebo can reduce the experienced disgust when viewing disgusting images“; „+ increase in brain activity in the frontal cortex“

S19 „Summary“

S20 „Placebos“; „= Inactive substances/procedures that cause people to feel better because they believe it will be so“; „1. Positive effects across a range of conditions/symptoms“; „2. Also influence behavior and physical processes (e.g., brain activation)“; „3. Can also work even when one knows they are taking a placebo“

S21 „Experiment“; „Testing of Open Label Placebos“

S22 „Experiment“; „Open Label Placebo in the form of a spray bottle filled with colored water“

S23 „Experiment“; „The placebo is intended to help you have less appetite."

S24 „We will show you 16 images."; „Smartphone: How much do you like the image? How much would you like to eat it right now?"

S25 „Example“

S26 …

S27 „Presentation for 6 seconds"

S28 „Smartphone: On a slider (0 to 100): How much do you like the picture? How much would you like to eat it right now?"

Supplementary Table S1: Open comments

Original (children)	Translation
1. "Mir hat das Placebo voll geholfen, ich habe gerade gar keinen Appetit mehr."	*"The placebo totally helped me, I have no appetite anymore."*
2. "Darf ich noch einmal?"	*"May I go again?"*
3. "Können wir das noch einmal machen?"	*"Can we do that again?"*
4. "Ich habe nichts gespürt."	*"I didn't feel anything."*
5. "Schmeckt grausig"	*"Tastes awful"*
6. "Ich habe jetzt weniger Appetit."	*"I have less appetite now."*
7. "Lecker schmeckt das."	*"This tastes delicious."*
8. "Ich habe gar keinen Hunger mehr."	*"I'm not hungry at all anymore."*
9. "Das schaut cool aus."	*"That looks cool."*
10. "Ich habe aber Hunger, das hilft ja gar nicht."	*"I am hungry, that doesn't help at all."*
11. "Ich habe jetzt wirklich keine Lust auf Essen."	*"I really don't feel like eating right now."*
12. "Bei mir hat es supidupi funktioniert."	*"It worked super-duper for me."*
13. "Das hat gar nicht gewirkt, ich bin noch hungriger als davor."	*"That didn't work at all, I'm even hungrier than before."*
14. "Ich habe überhaupt nichts gemerkt."	*"I didn't notice anything at all."*
15. "Bei mir hat es geholfen."	*"It has helped me."*
16. "Man merkt schon, dass es hilft, aber ist ja gar nicht so."	*"You can tell that it helps, but it's not really like that."*
17. "Ich glaube es hat sehr gut gewirkt."	*"I think it worked very well."*
18. "Ich hatte generell keinen Hunger."	*"I was generally not hungry."*

Original (adolescents)	Translation
1. "Mehr Hunger durch Placebo"	"More hunger due to placebo"
2. "Bei Panikattacken nehmen ich auch ein Placebo; hier hat es mir jetzt aber nicht so geholfen."	"I also take a placebo to treat panic attacks; but it didn't help me as much here."
3. „Ist wie Globuli“	“Is like globules”
4. "Ich habe dadurch mehr Hunger bekommen."	"It made me hungrier"
5. "Das hat mir jetzt nicht geholfen, aber ich kenne das von Prüfungsangst. Das hilft mir schon."	"That didn't help me now, but I know it from test anxiety. That helps me."
6. „Ist wie Homöopathie“	“Is like homeopathy”
7. "Ich glaube schon, dass es bei mir geholfen hat."	"I do believe it helped me."
8. "Meine Eltern haben mir früher immer einen Tee gemacht, der auch geholfen hat gegen Stress; dabei war gar nichts drinnen."	"My parents used to make me a tea that also helped with stress; there was nothing in it."
9. "Mit Einwirkzeit könnte es mir vielleicht geholfen haben, aber es ging dann ja direkt weiter."	"With more exposure time, it could have helped me, but then it went straight on."
10. "Ich habe Hunger - natürlich würde ich jetzt essen."	"I'm hungry - of course, I would eat now."
11. "Ich kann mir nicht vorstellen, dass das wirkt; aber wenn ich nicht wüsste, dass da nichts drinnen ist, dann würde das sicher viel besser wirken."	"I can't imagine that it works; but if I didn't know there was nothing in it,
12. "Ich glaube nicht das so etwas geht, denn das ist ja dumm uns das vorher zu sagen."	
13. "Ich glaube schon, dass so etwas funktionieren kann. Meine Mama hat mir als Kind auch immer einen Saft	

gegen Stress gemacht, von dem ich eigentlich wusste, dass eigentlich nichts drinnen ist; hat aber trotzdem funktioniert."<br>14. „Das ist umgekehrte Psychologie“	then it would surely work much better."<br><br>"I don't believe something like that works, because it's stupid to tell us beforehand."<br><br>"I do believe something like that can work. When I was a child, my mom used to make me a juice against stress, which I knew actually had nothing in it; but it still worked."<br><br>“This is reversed psychology”.
